# Blood glucose dynamics during sleep in patients with obstructive sleep apnea and normal glucose tolerance: effects of CPAP therapy

**DOI:** 10.1007/s11325-021-02442-9

**Published:** 2021-08-11

**Authors:** Kimimasa Saito, Yosuke Okada, Keiichi Torimoto, Yoko Takamatsu, Yoshiya Tanaka

**Affiliations:** 1 Saito Naika Kokyukika, Mie Sleep Clinic, 446 Sogo, Obata-chyo, Ise-shi, Mie 519-0502 Japan; 2 First Department of Internal Medicine, School of Medicine, University of Occupational and Environmental Health, Kitakyushyu-shi, 807-8555 Japan

**Keywords:** Obstructive sleep apnea, Continuous glucose monitoring, Flush glucose monitoring, Glycemic variability during sleep, Continuous positive airway pressure, REM-related obstructive sleep apnea

## Abstract

**Purpose:**

Glycemic variability (GV) and hypoglycemia during nighttime are presumed to be associated with fatal bradycardia. The aim of this prospective study was to evaluate blood glucose dynamics during sleep in patients with obstructive sleep apnea syndrome (OSA) and normal glucose tolerance.

**Methods:**

Patients with OSA and no diabetes who underwent type 1 overnight polysomnography from December 2018 to May 2020 participated in this study. GV was evaluated in all participants for 14 days using a flash glucose monitoring device. Correlations were examined between GV indexes and indexes related to sleep breathing disorders, the effects of treatment with continuous positive airway pressure (CPAP) on these GV indexes, and the characteristics of glucose dynamics in different OSA subtypes classified by sleep stage.

**Results:**

Among 42 patients with OSA and no diabetes, the standard deviation of GV during sleep correlated significantly with sleep time spent with oxygen saturation <90% (*r*=0.591, *p*=0.008). High blood glucose index during sleep correlated significantly with stage N1% (*r*=0.491, *p*=0.032) and negatively with stage N2% (*r*=−0.479, *p*=0.038). High blood glucose index correlated significantly with sleep time spent with oxygen saturation <90% (*r*=0.640, *p*=0.003). The rapid eye movement–related OSA group had a higher incidence of hypoglycemia. One-week with CPAP treatment significantly improved GV during sleep, standard deviation of GV (from 12.1 to 9.0 mg/dL, *p*<0.001), and high blood glucose index (from 0.7 to 0.4, *p*=0.006).

**Conclusions:**

To evaluate GV during sleep in patients with OSA may be useful for clinical risk management. CPAP treatment for 1 week may have an improving GV and high blood glucose index.

**Clinical trial registration:**

UMIN000038489 2019/11/04, UMIN 000025433 2016/12/27

**Supplementary Information:**

The online version contains supplementary material available at 10.1007/s11325-021-02442-9.

## Introduction

In patients with obstructive sleep apnea (OSA) syndrome, progression of arteriosclerosis is enhanced by increased sympathetic nerve activity associated with nocturnal awakening and sleep fragmentation [1], vascular endothelial dysfunction via inflammation and oxidative stress associated with intermittent hypoxia [2], and increased arterial stiffness [3]. In contrast, in type 2 diabetes mellitus (T2DM), increased glycemic variability (GV) is considered to play a part in the onset and progression of macroangiopathy [4], and GV has been linked to the risk of death associated with cardiovascular events [5]. Research studies suggest that postprandial hyperglycemia may be involved in the onset of microangiopathy and can be used as a predictor of myocardial infarction [6–8]. Moreover, hypoglycemia has been reported to be associated with cardiovascular risk and increased risk of death in patients with T2DM [9, 10]. We have previously reported that GV, hyperglycemia, and hypoglycemia are probably involved in the pathophysiology of vascular endothelial dysfunction in patients with T2DM [11]. In this regard, latest advances in T2DM treatment suggest the need for more than just the monitoring of average blood glucose level using glycated hemoglobin (HbA1c) values and call for high-quality glycemic control with minimal GV.

Gami et al. reported that sudden death in patients with OSA during nighttime sleep might be associated with life-threatening arrhythmia and ischemic heart disease [12]. The involvement of GV and hypoglycemia during sleep is presumed to be a contributing factor in these events. One study suggested that asymptomatic hypoglycemia is more likely to occur during the night, including the time of sleep, and induce bradyarrhythmia [13]. In the worst-case scenario, it can lead to “dead in bed syndrome” [14]. However, only a few studies have investigated glycemic variability during sleep in patients with OSA. Most of the past studies explored the association between within-day GV and apnea/hypopnea index (AHI) in diabetic patients with OSA. On the other hand, CPAP treatment has previously been shown to improve other hemodynamic parameters in patients with OSA such as heart rate variability [15]. It is clinically important to carefully monitor glycemic variability during sleep and confirm the effect of CPAP treatment on glycemic variability in patients with OSA. However, limited studies have reported the details of blood GV during sleep in patients with OSA who have normal glucose tolerance.

Therefore, the purpose of this study was to fill this gap in our understanding of blood glucose dynamics in sleeping patients with OSA. To achieve this, we monitored GV during sleep in OSA patients without diabetes. Further data analysis elucidated the correlations of GV indexes with indexes related to sleep-disordered breathing obtained by overnight polysomnography (PSG); examined the effects of treatment with continuous positive airway pressure (CPAP) on these GV indexes; and compared the characteristics of glucose dynamics in different OSA subtypes classified based on sleep stage.

## Methods

### Participants

The present prospective observational study included patients who visited Mie Sleep Clinic, Mie, Japan, between December 1, 2018, and May 31, 2020. Patients admitted for overnight PSG who were not on treatment for T2DM were included. All the patients provided written informed consent after we provided information regarding the study procedure. This study was approved by the Institutional Review Board (IRB) of Medical Corporation MSC (#17001, UMIN000038489) and University of Occupational and Environmental Health School of Medicine (Trial registration: H27-186, Registered 25 Dec 2015, UMIN 000025433) and was carried out in accordance with the Declaration of Helsinki.

### Study design

All participants were asked to wear a flash glucose monitoring (FGM) device (FGMS® System FreeStyle Libre Pro System, Abbott Diabetes Care, Inc.) (day 0). A week later, blood samples were collected for evaluation of blood glucose level. Patients suspected of OSA also underwent overnight PSG on admission and those who met the criteria listed below were introduced to CPAP treatment at day 7. At the end of the 2-week study period, the FGM device was collected from all the subjects (day 14), and the CPAP devices were checked for nighttime use and indexes of OSA. We compared the FGM data corresponding to days 1–7 (6 nights) and 8–13 (6 nights) as the data of pre-CPAP and post-CPAP observation periods, respectively (see Fig. S1 in the supplemental material).

Participants were asked to restrict their food intake except for water for 2 h before bedtime and log the time at which they ate, went to bed, and woke up, in an action table. Fluctuations in glucose levels from bedtime to the time of waking up, as derived from the logs in the action table, were defined as GV during sleep.

The study included patients who met the following criteria: (1) age of 20–80 years at the time of provision of informed consent, (2) AHI of ≥15/h on PSG, and (3) absence of diagnosis or treatment of T2DM prior to the admission. We excluded patients who met the following criteria: (1) AHI of <15/h, (2) failure to record FGM values throughout the observation periods until day 7, and (3) difference of ≥20 mg/dL between the measured blood glucose level and recorded FGM value at almost the same time. For patients with average CPAP usage time of <4 h, we used their pre-CPAP data only.

The control group included healthy individuals without OSA (based on history, including lack of snoring and witnessed apnea during sleep) who had normal glucose tolerance. They underwent FGM 24-h data recording for 2 weeks and these data were used for comparison with patients with OSA.

The primary endpoint was to evaluate the correlation between various indexes of GV during sleep and indexes of sleep-disordered breathing, and to examine changes in GV during sleep in patients with OSA after CPAP treatment. The secondary endpoint was comparison of characteristics of glucose dynamics in different OSA subtypes classified based on sleep stage.

### Flash glucose monitoring system

The average glucose level (AG), standard deviation (SD), coefficient of variation (CV), percentage of time at which glucose level was between 70 and 180 mg/dL (TIR, time in range), percentage of time at which glucose level was <70 mg/dL (TBR, time below range), percentage of time at which glucose level was ≧ 180 mg/dL (TAR, time above range) [16], low blood glucose index (LBGI), and high blood glucose index (HBGI) were calculated from the data recorded using an FGM device [17, 18]. Hypoglycemia was defined as interstitial glucose level of <70 mg/dL, as measured by FGM. FGM data were recorded for 14 consecutive days from the second day, excluding the first day when the sensor was attached to avoid any bias related to the insertion and removal of FGM or insufficient stability of the monitoring system. We obtained the daily average value and listed the average value for six nights.

### Polysomnography

Full attended overnight PSG was conducted in a laboratory setting, which was classified as type 1 sleep study by the American Academy of Sleep Medicine (AASM). PSG was performed using the Alice® 6 LDx system (Philips Respironics, Murrysville, PA). Sleep and respiratory event scoring were carried out according to the AASM manual ver. 2.4. 1 [19]. To achieve this, the digital sleep/breathing record was analyzed for the absolute and relative durations of each sleep stage, total sleep time (TST), sleep efficiency, and wake after sleep onset. Arousals were scored according to the AASM criteria, with an arousal index (ArI) calculated as the number of arousals per hour of sleep. Apneas were defined relative to the baseline as >90% decrease in airflow for >10 s; hypopneas were defined as reduction in airflow signal (typically >30% decrease, accompanied by inspiratory flow limitation) for >10 s, associated with or without O_2_ desaturation and/or EEG-defined arousals. Thus, hypopneas were separated into those with 3% desaturation and others with EEG arousal only. TST, percentage of sleep time spent with oxygen saturation <90% (SLT90), ArI, AHI, AHI during rapid eye movement (REM) sleep (REM-AHI), AHI during non-REM (NREM) sleep (NREM-AHI), and the oxygen desaturation index (ODI) were calculated from the data recorded through PSG.

### Continuous positive airway pressure

Patients diagnosed with moderate or severe OSA were advised to commence CPAP treatment as a part of standard care for OSA. Patients who agreed to undergo CPAP therapy were provided with an auto-titrating device (Dream Station Auto: Philips Respironics). All participating patients received usual care at the outpatient clinics. After 1 week, the usage data were downloaded from the CPAP machine.

### OSA subtype classification

Based on AHI by sleep stage, the OSA group was further divided into two groups: (1) the REM-related OSA group (AHI in REM/AHI in NREM >2) and (2) the non-specific OSA group (AHI in REM/AHI in NREM ≤2) [20].

### Statistical analysis

Values are expressed as mean±standard deviation. The Shapiro–Wilk test was used to test data distribution. Student’s *t*-test was used to differences between two groups with normally distributed data and equal variance as confirmed by the *F* test. Welch’s *t*-test was used for other normally distributed data with unequal variance. The Mann–Whitney *U* test was used for comparison of two groups with skewed data distribution. The Spearman’s rank correlation coefficient was used to analyze the relationship between two variables. Probability values ≤0.05 were considered significant. All statistical analyses were performed with EZR Ver.1.52 (Saitama Medical Center, Jichi Medical University, Saitama, Japan), which is a graphical user interface for the R version 4.02 (The R Foundation for Statistical Computing, Vienna, Austria). Specifically, it is a modified version of the R commander designed to add statistical functions frequently used in biostatistics.

## Results

### Baseline characteristics

Figure S2, in the supplemental material, shows a flow diagram of the patient selection process. A total of 65 (15 healthy individuals and 50 patients with suspected OSA) provided consent to participate in this study. Two of the 15 healthy individuals had missing FGM data during recording, and 2 of the 50 individuals who received PSG for suspected OSA had AHI of <15/h. Among the 48 patients who underwent CPAP therapy, 4 had missing FGM data, 2 had a difference of ≥20 mg/dL between the actual and FGM-based blood glucose levels, and 3 had an average CPAP usage of <4 h during the first week after CPAP initiation. The baseline characteristics of the 42 patients with OSA (25 men) are shown in Table 1.

**Table 1:** Baseline characteristics

Variables	OSA (*n* = 42)
Sex (male/female)	25/17
Age, years	52.7 ± 11.8
Height, cm	163.6 ± 10.0
Weight, kg	79.3 ± 24.2
BMI, kg/m^2^	29.4 ± 7.7
Blood pressure (mm Hg)	
SBP	127.5 ± 10.1
DBP	78.5 ± 8.3
Biochemical indicators	
Glucose (mg/dL)	106.2 ± 23.9
1,5AG (μg/dL)	19.2 ± 8.0
LDL cholesterol (mg/dL)	122.4 ± 27.9
Triglycerides (mg/dL)	212.4 ± 189.8
AST (IU/L)	28.6 ± 17.2
ALT (IU/L)	38.6 ± 30.7
BUN (mg/dL)	14.5 ± 3.2
Creatinine (mg/dL)	0.77 ± 0.2
eGFR (Cr) (mL/min/1.73m^2^)	77.8 ± 16.7
Comorbidities, *n* (%)	
HT	11 (26.2)
CHD	1 (2.4)
CI	3 (7.1)
MetS	18 (42.9)

Data are presented as mean ± SD

*OSA* obstructive sleep apnea, *BMI* body mass index, *SBP* systolic blood pressure, *DBP* diastolic blood presure, *HT* hypertension, *CHD* coronary heart disease, *CI* cerebral infarction, *MetS* metabolic syndrome

### FGM variables

Table 2 shows the baseline FGM data of the 42 patients with OSA during sleep and day (24 h). The comparison of FGM indexes between normal participants (*n*=13) and patients with OSA (*n*=42) is shown in Table S1 in the supplemental material. Since the number of cases in normal participants is small, this data is for reference only. The GV indexes, such as SD, CV, and HBGI, in the OSA group tended to be higher than those in the control group.

**Table 2:** FGM indexes of patients with OSA (*n*=42)

	OSA (*n* = 42)
FGM indexes during sleep	
AG, mg/dL	100.3 ± 19.5
SD, mg/dL	12.0 ± 5.4
CV, %	11.9 ± 4.0
TBR, %	5.8 ± 11.7
TIR, %	91.9 ± 12.6
TAR, %	1.1 ± 3.6
LBGI	2.5 ± 2.4
HBGI	0.7 ±1.0
Magnitude of the dawn phenomenon, mg/dL	17.2 ± 8.3
FGM indexes during 24 h	
AG, mg/dL	113.2 ± 19.2
SD, mg/dL	25.0 ± 8.5
CV, %	22.0 ± 5.5
TBR, %	2.7 ± 4.5
TIR, %	91.9 ± 8.2
TAR, %	4.9 ± 8.2
LBGI	2.0 ± 1.5
HBGI	2.3 ± 1.8

Data are presented as mean ± SD

*FGM* flash glucose monitoring system, *OSA* obstructive sleep apnea, *AG* average glucose, *SD* standard deviation, *CV* coefficient of variation, *TBR* time below range (percentage of time for which glucose level was <70 mg/dL), *TIR* time in range (percentage of time for which glucose level was 70–180 mg/dL), *TAR* time above range (percentage of time for which glucose level was >180 mg/dL), *LBGI* low blood glucose index, *HBGI* high blood glucose index

### Relationship between parameters of PSG and FGM during sleep (*n*=42)

**Fig. 1:**
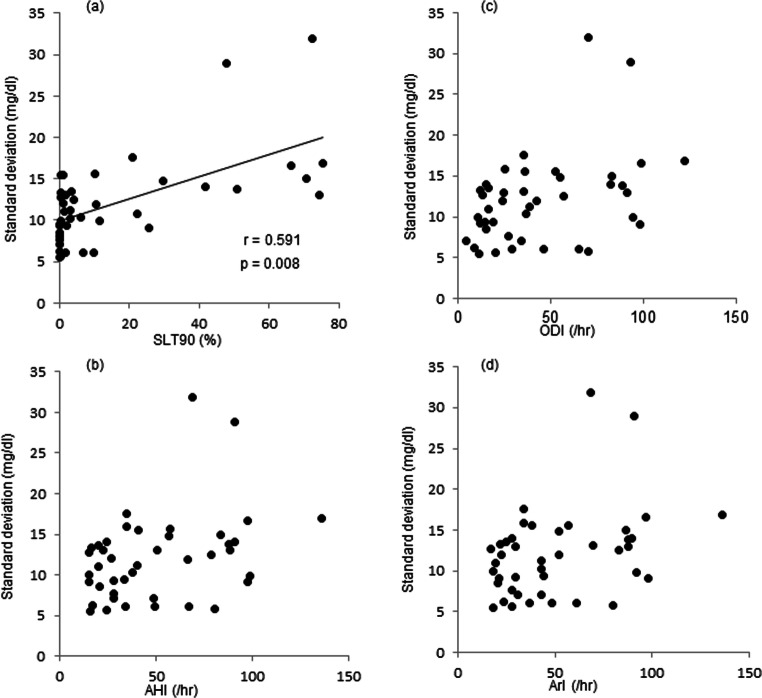
Relationship between indexes of PSG and standard deviation (SD) of FGM during sleep. SD data of individual patients are plotted against their SLT90 (**a**), AHI (**b**), ODI (**c**), and ArI (**d**). Note the significant correlation between SD and SLT90 (*y*=0.1343*x* + 9.9338, *r*=0.591, *p*=0.008). SLT90, time spent with oxygen saturation at <90%; AHI, apnea/hypopnea index; ODI, oxygen desaturation index; ArI, arousal index

Table S2 (in the supplemental material) shows the PSG data of the 42 patients of the OSA group, and Table 3 shows the correlations between PSG and FGM indexes during sleep. SD during sleep correlated significantly with SLT90 only (*r*=0.591, *p*=0.008; Fig. 1) and tended to increase with increase in the severity of OSA.

**Table 3:** Correlations between indexes of polysomnography and indexes of FGM during sleep in patients with OSA (*n*=42)

			AG	SD	CV	TBR	TIR	TAR	LBGI	HBGI	MDP
Sleep stage	TST	*R*	0.138	0.179	0.055	−0.240	−0.239	0.148	−0.141	0.103	0.202
		*p* value	0.574	0.462	0.822	0.323	0.324	0.545	0.565	0.676	0.406
	REM sleep time/TST	*R*	0.066	0.035	−0.074	−0.274	−0.219	−0.001	−0.177	−0.026	0.173
		*p* value	0.788	0.887	0.764	0.256	0.368	0.998	0.470	0.915	0.478
	N1 stage time/TST	*R*	0.462	0.434	0.330	−0.223	0.133	0.428	−0.336	0.491	0.02
		*p* value	0.046	0.063	0.168	0.359	0.586	0.067	0.160	0.032	0.992
	N2 stage time/TST	*R*	−0.454	−0.423	−0.306	0.284	−0.155	−0.440	0.343	−0.479	−0.012
		*p* value	0.051	0.071	0.203	0.238	0.527	0.059	0.150	0.038	0.960
	N3+4 stage time/TST	*R*	−0.377	−0.335	−0.214	0.122	−0.128	−0.176	0.354	−0.311	−0.147
		*p* value	0.112	0.160	0.377	0.620	0.371	0.471	0.137	0.195	0.478
Severity of OSA	AHI total	*R*	0.452	0.356	0.224	−0.267	0.139	0.471	−0.319	0.498	−0.009
		*p* value	0.052	0.135	0.357	0.269	0.570	0.041	0.184	0.030	0.971
	REM-AHI	*R*	−0.010	−0.069	−0.134	0.004	−0.081	0.120	0.244	0.073	−0.180
		*p* value	0.966	0.663	0.586	0.989	0.740	0.626	0.315	0.765	0.463
	NREM-AHI	*R*	0.464	0.347	0.203	−0.266	0.143	0.472	−0.327	0.502	−0.011
		*p* value	0.045	0.145	0.404	0.271	0.558	0.041	0.172	0.028	0.964
	ArI	*R*	0.449	0.359	0.233	−0.261	0.149	0.478	−0.324	0.498	0.025
		*p* value	0.054	0.131	0.336	0.280	0.544	0.039	0.176	0.030	0.918
	ODI	*R*	0.443	0.405	0.278	−0.263	0.131	0.475	−0.302	0.508	−0.002
		*p* value	0.057	0.085	0.250	0.276	0.592	0.041	0.208	0.027	0.995
	SLT90	*R*	0.475	0.591	0.477	−0.261	0.122	0.517	−0.310	0.640	0.149
		*p* value	0.040	0.008	0.039	0.281	0.618	0.023	0.196	0.003	0.544

Data are results of Spearman’s correlation analysis

*FGM* flash glucose monitoring system, *OSA* obstructive sleep apnea, *TST* total sleep time, *REM* rapid eye movement, *AHI* apnea/hypopnea index, *NREM* non-rapid eye movement, *ArI* arousal index, *ODI* oxygen desaturation index, *SLT90* percentage of sleep time spent with oxygen saturation <90%, *AG* average glucose, *SD* standard deviation, *CV* coefficient of variation, *TBR* time below range (percentage of time for which glucose level was <70 mg/dL), *TIR* time in range (percentage of time for which glucose level was 70–180 mg/dL), *TAR* time above range (percentage of time for which glucose level was >180 mg/dL), *LBGI* low blood glucose index, *HBGI* high blood glucose index, *MDP* magnitude of the dawn phenomenon

**Fig. 2:**
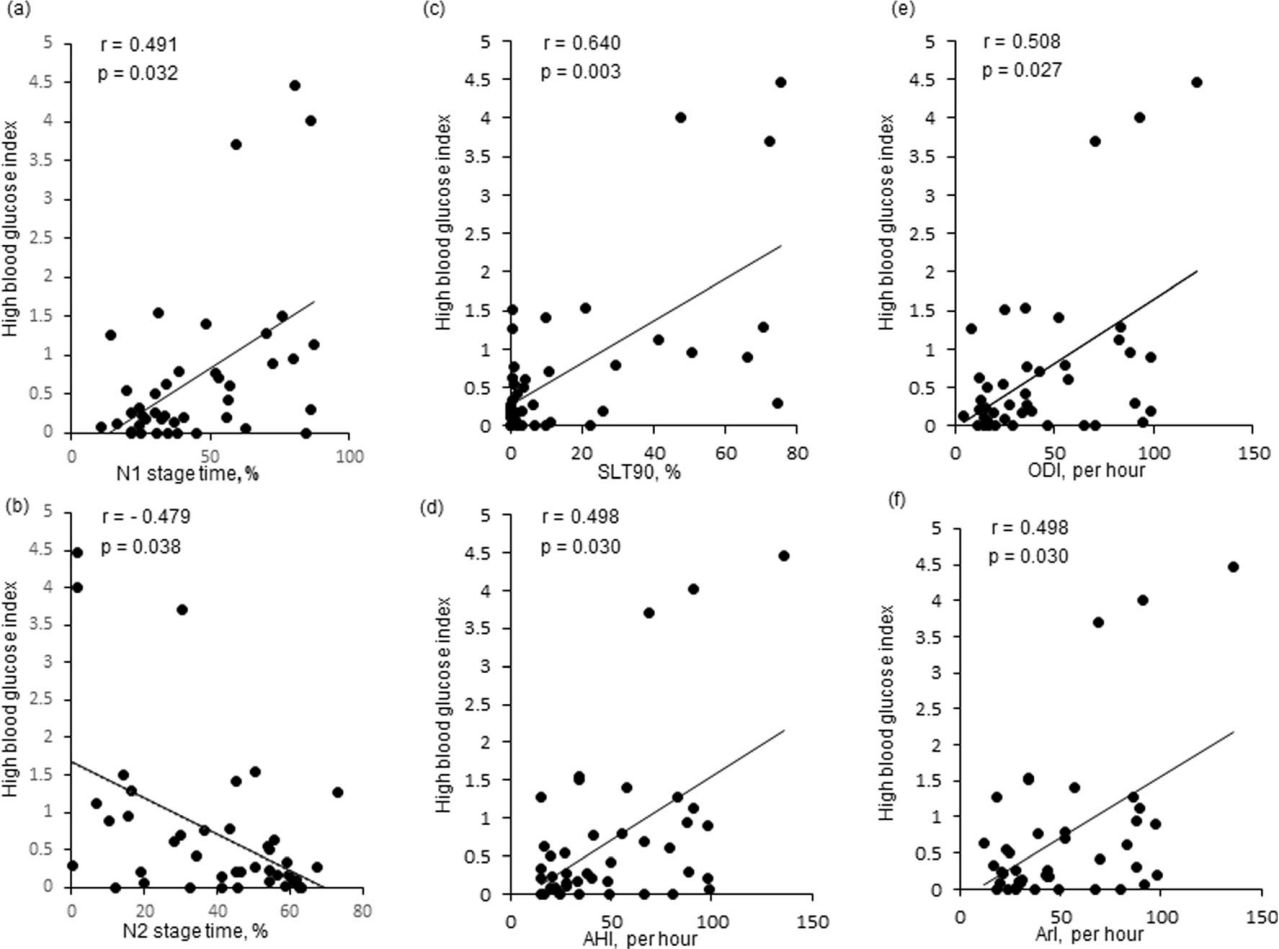
Relationship between sleep indexes of PSG and high blood glucose index of FGM. High blood glucose index (HBGI) data of individual patients are plotted against N1 stage time % (**a**), N2 stage time% (**b**), SLT90 (**c**), AHI (**d**), ODI (**e**), and ArI (**f**). Among these relationships, the correlation between HBGI and SLT90 was the strongest (*y*=0.0274*x* + 0.2681, *r*=0.640, *p*=0.003). SLT90, time spent with oxygen saturation at <90%; AHI, apnea/hypopnea index; ODI, oxygen desaturation index; ArI, arousal index

HBGI during sleep correlated significantly with stage N1% (*r*=0.491, *p*=0.032) and negatively with stage N2% (*r*=−0.479, *p*=0.038) (Fig. 2). With regard to the severity of OSA, HBGI correlated significantly with AHI (*r*=0.498, *p*=0.030), NREM-AHI (*r*=0.502, *p*=0.028), ArI (*p*=0.498, *p*=0.030), ODI (*r*=0.508, *p*=0.027), and SLT90 (*r*=0.640, *p*=0.003) (Fig. 2).

TAR during sleep also correlated significantly with the severity of OSA, based on its correlation with AHI (*r*=0.471, *p*=0.041), NREM-AHI (*r*=0.472, *p*=0.041), ArI (*r*=0.478, *p*=0.039), ODI (*r*=0.475, *p*=0.041), and SLT90 (*r*=0.517, *p*=0.023). In particular, GV tended to be high in OSA patients with high incidence of sleep-disordered breathing events during NREM stage and high percentage of N1 stage.

### REM-OSA versus non-stage-specific OSA (*n*=19 vs *n*=23)

**Fig. 3:**
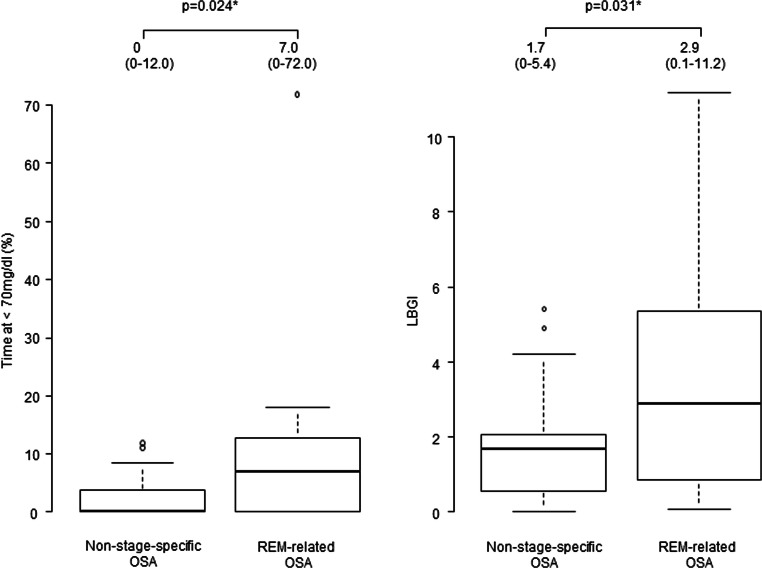
Comparison of time below range and risk indexes of glucose variability: non-stage-specific OSA group vs. REM-related group. The REM-related OSA group spent a significantly longer time at a glucose level below 70 (*p* = 0.024) and LBGI (*p* = 0.031) during sleep than did the non-stage-specific OSA group. In these box-and-whisker plots, lines within the boxes represent median values; the upper and lower lines of the boxes represent the 25th and 75th percentiles, respectively; and the upper and lower bars outside the boxes represent the 90th and 10th percentiles, respectively. *p≤0.05, by the Mann–Whitney *U* test. REM, rapid eye movement; LBGI, low blood glucose index

Based on the significant differences in GV among sleep stages, we examined the characteristics of GV according to the OSA subtypes. As shown in Fig. 3, TBR (time at below 70 mg/dL) and LBGI (*p*=0.024 and 0.031, respectively) were significantly higher in the REM-related OSA group than those in the non-specific OSA group.

### Effects of CPAP treatment on FGM parameters (*n*=39)

**Fig. 4:**
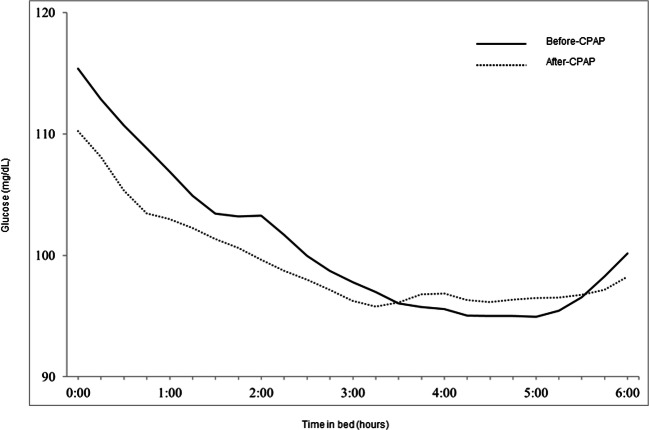
Average glucose levels measured using flash glucose monitoring device. Average glucose levels recorded by flash glucose monitoring before CPAP treatment (solid line) and 1-week time point after CPAP treatment (dotted line) in patients with OSA

Figure 4 and Table 4 summarize the effects of CPAP treatment on various FGM indexes during sleep. While 1-week CPAP treatment had no effect on AG during sleep, it significantly improved all other indexes of GV, such as SD (from 12.1 to 9.0 mg/dL, *p*<0.001), CV (from 11.7 to 8.9%, *p*<0.001), TIR (from 92.4 to 97.1%, *p*=0.003), and magnitude of the dawn phenomenon (MDP) (from 17.7 to 12.0 mg/dL, *p*<0.001). Furthermore, CPAP treatment significantly improved HBGI (from 0.7 to 0.4, *p*=0.006) and TBR (from 6.4 to 2.5%, *p*=0.011), representing the risk of hyperglycemia and hypoglycemic time, respectively.

**Table 4:** FGM and sleep indexes of patients treated with CPAP (*n* = 39)

Indexes	Before	After	*p* value
Sleep indexes			
Mean sleep time, h	6.6 ± 0.6	6.5 ± 0.6	0.310
CPAP usage time, h/night	-	5.5 ± 1.2	-
AHI, no./h	50.6 ± 30.5	4.1 ± 2.7	<0.001^*^
FGM indexes during sleep			
AG, mg/dL	101.0 ± 19.8	99.1 ± 16.8	0.372
SD, mg/dL	12.1 ± 5.5	9.0 ± 4.5	<0.001*
CV, %	11.7 ± 4.1	8.9 ± 3.6	<0.001*
TBR, %	6.4 ± 12.5	2.5 ± 6.9	0.011*
TIR, %	92.4 ± 12.6	97.1 ± 7.0	0.003*
TAR, %	1.2 ± 3.8	0.4 ± 1.1	0.111*
LBGI	2.5 ± 2.5	2.0 ± 1.9	0.058
HBGI	0.7 ± 1.1	0.4 ± 0.7	0.006*
Magnitude of the dawn phenomenon, mg/dL	17.7 ± 8.3	12.0 ± 7.5	<0.001*
FGM indexes during 24h			
AG, mg/dL	114.4 ± 19.2	108.9 ± 23.8	0.051
SD, mg/dL	25.2 ± 8.7	23.6 ± 8.2	<0.001*
CV, %	22.0 ± 5.7	21.1 ± 5.7	0.028*
TBR, %	2.7 ± 4.6	2.3 ± 4.5	0.416
TIR, %	92.2 ± 8.7	93.7 ± 8.7	0.092
TAR, %	5.2 ± 8.4	4.1 ± 8.0	0.175
LBGI	1.9 ± 1.5	1.7 ± 1.3	0.249
HBGI	2.3 ± 1.9	2.0 ± 1.7	0.017*

Data are presented as mean ± SD. Paired *t*-tests were used to compare before and after CPAP treatment outcomes. *p values ≤0.05 were considered significant

*FGM* flash glucose monitoring system, *CPAP* continuous positive airway pressure, *OSA* obstructive sleep apnea, *AHI* apnea/hypopnea index, *AG* average glucose, *SD* standard deviation, *CV* coefficient of variation, *TBR* time below range (percentage of time for which glucose level was <70 mg/dL), *TIR* time in range (percentage of time for which glucose level was 70–180 mg/dL), *TAR* time above range (percentage of time for which glucose level was >180 mg/dL), *LBGI* low blood glucose index, *HBGI* high blood glucose index, *MDP* magnitude of the dawn phenomenon

## Discussion

Our study demonstrated significantly greater GV, evidenced by nocturnal values of SD, CV, TIR, and HBGI, in patients with OSA and normal glucose tolerance, compared with healthy individuals. The results also suggested possible increase in various markers of hypoglycemia in patients with REM-related OSA. In addition, various indexes of OSA severity, particularly percent time in hypoxemia, were associated with nocturnal GV. Our results also showed that 1-week CPAP treatment significantly improved nocturnal GV in patients with OSA. We consider it particularly important to evaluate GV during sleep in patients with OSA.

Peng et al. compared GV between patients with non-diabetic OSA (*n*=86) and healthy controls (*n*=40). They showed that the OSA group had significantly higher levels of postprandial blood glucose and within-day and nighttime mean amplitude of glycemic excursion (MAGE) than the control group and concluded that OSA severity (AHI) was positively correlated with these GV indicators [21].

Nakata et al. reported a positive correlation between log MAGE and AHI [22], and concluded that AHI negatively affects GV. Kurosawa et al. found a significant and positive correlation between AHI and NREM-AHI with SD [23], another index of GV. Khaire et al. reported that night CV% and MAGE between 10 pm and 6 am were significantly higher in 10 patients with OSA compared with those in 10 patients without OSA [24].

In the present study, the values of various markers of GV (e.g., SD and CV) during sleep, in addition to TIR, HBGI, and MDP, were higher in patients with OSA than those in the healthy control group.

We also examined the associations between various indices related to breathing disorders and markers of GV during sleep. The results showed worsened GV (SD) and hyperglycemic risk (HGBI) with increased severity of OSA, especially with the percent time in hypoxemia (SLT90), rather than with AHI.

What is the underlying mechanism(s) of GV during sleep in non-diabetic patients with OSA? While our study did not directly examine these mechanisms, previous studies hinted to the potential roles of hypoxemia. In this regard, Hui et al. reported that the more pronounced the sleep-related hypoxemia is in patients with OSA and T2DM, the higher the likelihood of hyperglycemia [25]. Their results also suggested the presence of mechanisms that increase oxidative stress and catecholamine release. Sun et al. used experimental animals to show that acute hypoxia promoted glycogenolysis and glycolysis in the liver and consequently increased plasma glucose levels, suggesting that energy metabolism is defined by the degree and duration of hypoxemia [26]. It is possible that the observed GV in our patients with OSA and normal glucose tolerance is related to one or more of the above effects of acute hypoxemia on glucose metabolism. Further clinical and experimental studies are needed to determine the true mechanism of OSA-related changes in glucose metabolism.

Several studies have demonstrated the beneficial effects of CPAP therapy in patients with T2DM. Morariu et al. studied 23 patients with OSA and T2DM were divided into using active CPAP group and sham CPAP group for 30 days. Active CPAP therapy demonstrated improvements in glycemic control as determined using serum fructosamine but not coutinuous glucose monitoring [27]. Another study reported improvement in blood glucose fluctuations after CPAP therapy [28, 29]. The above studies were conducted in patients with T2DM, and CPAP therapy was considered to have contributed to improvement in insulin resistance, which was considered the primary factor contributing to these improvements. In contrast to the above studies, another study of 13 patients with OSA free of T2DM showed no significant improvement in HOMA-IR after 2.5 years of CPAP therapy despite improvements in GV [22]. Unlike the preceding studies, the latter study focused on GV in patients with OSA and normal glucose tolerance. Similarly, our study was conducted in patients with normal glucose tolerance and we also showed marked improvement in GV after 1 week of CPAP therapy.

In this study, we further found that the risks of hypoglycemia, such as TBR and LBGI, were significantly higher in the REM-related OSA group than those in the non-specific OSA group. In contrast, the risk of hyperglycemia was positively correlated with NREM-AHI, arousal index, and percentage of stage N1. What are the mechanisms of hyperglycemia or hypoglycemia risks increase even during sleep? Regarding the risks of hyperglycemia, hypoxemia and the resultant sleep fragmentation [1] are important factors. The assumed mechanism is that both conditions can lead to extreme hypertonicity of the sympathetic nervous system, which stimulates the glycolytic system. Regarding the risks of hypoglycemia in REM-related OSA, the mechanism is probably related to the enhanced dominance of the parasympathetic activities during NREM sleep by counter adjustment of the autonomic nervous system and the insufficient prevention of hypoglycemia due to the suppressed flash secretion of growth hormones [30]. Jones et al. reported possible nocturnal counter-regulatory hormone responses even in healthy individuals [31]. Furthermore, Bialasiewicz et al. reported that the early morning fall in blood glucose levels is reversed by sleep breathing disorders during the REM sleep, presumably due to the involvement of altered neuroendocrine regulation [32]. Since the longest phase of REM sleep occurs in the latter part of the sleep cycle, patients with typical REM-related OSA experience relatively long and frequent apneas during the early morning hours compared to fewer apneas in the first few hours of sleep. Based on the compensatory decrease in epinephrine secretion due to counter-regulatory hormone responses, parasympathetic nerves become relatively dominant in the first part of sleep, leading to a decrease in glucagon secretion or downregulation of other pathways, possibly resulting in falls in blood glucose levels. Furthermore, in this study, many patients did not reach stage N3. Therefore, there was a possibility that the hypoglycemia prevention mechanism did not work due to the suppressed flash secretion of growth hormones [30].

The results of our study also suggest that the mechanism of glycemic fluctuations during sleep in patients with OSA involves catecholamine-mediated effects induced through acute hypoxemia and/or counter-regulatory hormone responses, rather than altered insulin sensitivity.

There are some limitations to this study. First, the diet of each patient was not standardized because we enrolled outpatients. Second, the sample size in the present study was relatively small. In particular, the number of control participants was small, and PSG testing was not performed. Our results should be verified in another study that includes a larger number of subjects.

In conclusion, the results of the present study demonstrated greater GV during sleep in patients with OSA and normal glucose tolerance, compared with healthy individuals. In addition, GV during sleep and the risk of hyperglycemia increased with the severity of OSA. Furthermore, several indexes of GV during sleep improved significantly after 7-day CPAP therapy. On the other hand, our results demonstrated that patients with REM-related OSA are potentially at higher risk of developing hypoglycemia.

## Supplementary Information

ESM 1 (TIF 69 kb)

High resulotion (PNG 5 kb)

ESM 2 (TIF 77 kb)

High resulotion (PNG 7 kb)

ESM 3 (DOC 1947 kb)
