# Supplementary figures and images for: Blood glucose dynamics during sleep in patients with obstructive sleep apnea and normal glucose tolerance: effects of CPAP therapy
Source: Sleep Breath. 2021 Aug 11;26(2):771–81. doi: 10.1007/s11325-021-02442-9 (PMC9130196; doi:10.1007/s11325-021-02442-9)

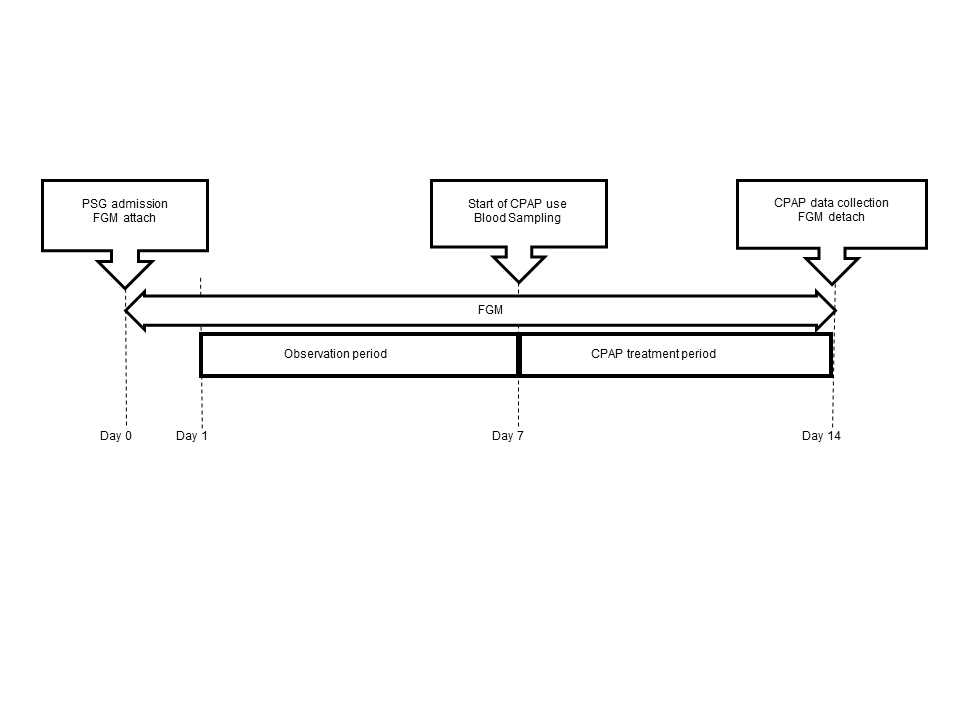

Supplement: Supplementary file 1 — (TIF 69 kb) [file 11325_2021_2442_MOESM1_ESM.tif]

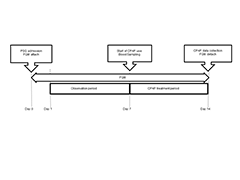

Supplement: Supplementary file 2 — High resulotion (PNG 5 kb) [file 11325_2021_2442_Fig5_ESM.png]

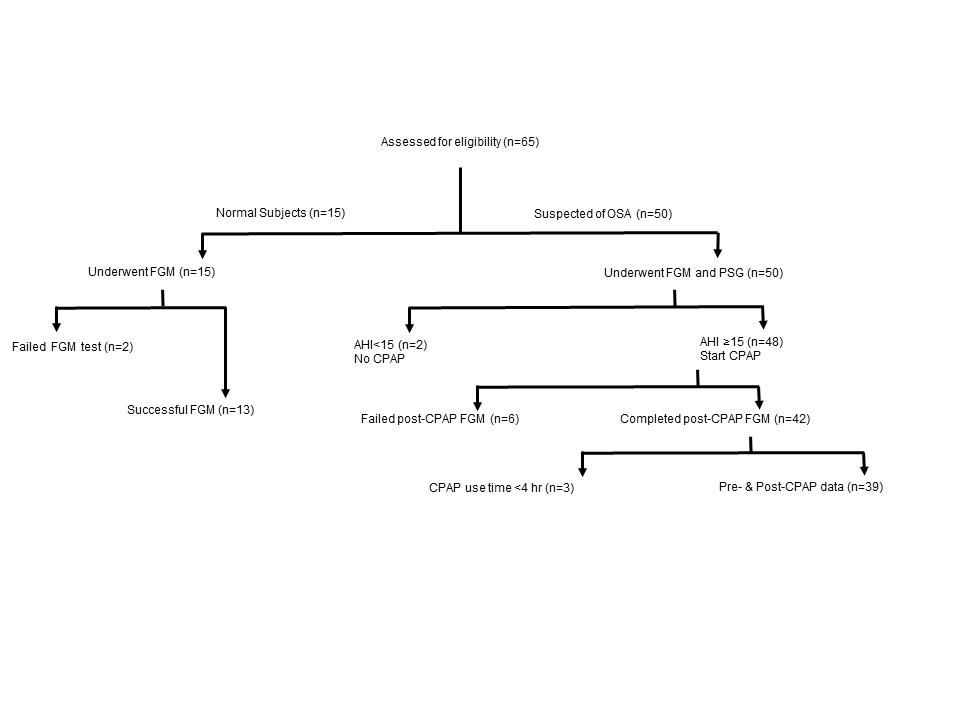

Supplement: Supplementary file 3 — (TIF 77 kb) [file 11325_2021_2442_MOESM2_ESM.tif]

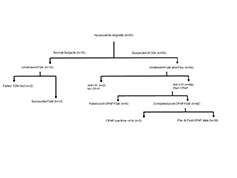

Supplement: Supplementary file 4 — High resulotion (PNG 7 kb) [file 11325_2021_2442_Fig6_ESM.png]
