# Supplementary material for: Blood glucose dynamics during sleep in patients with obstructive sleep apnea and normal glucose tolerance: effects of CPAP therapy
Source: Sleep Breath. 2021 Aug 11;26(2):771–81. doi: 10.1007/s11325-021-02442-9 (PMC9130196; doi:10.1007/s11325-021-02442-9)
Supplement: Supplementary file 5 — (DOC 1947 kb) [file 11325_2021_2442_MOESM3_ESM.doc]

**Supplementary information**

Supplemantal Table S1

Supplemental Table S2

Supplemental Figure S1

Supplemental Figure S2

**Figure Legends**

Supplemental Fig. S1 Outline of the study protocol

On day 0, patients with suspected obstructive sleep apnea (OSA) were admitted to the hospital for the over-night polysomnography (PSG) test, and they were asked to wear a flash glucose monitoring (FGM) device. On day 7, blood samples were collected for evaluation of blood glucose level, and continuous positive airway pressure (CPAP) was introduced at the outpatient department. On day 14, data were collected from the CPAP device and FGM was detached. The observation period was from the night of day 1 to the night of day 6, and the CPAP treatment period was from the night of day 7 to the night of day 13

Supplemental Fig. S2 Flow diagram of the study subject selection process

FGM, flash glucose monitoring system; PSG, polysomnography; AHI, apnea/hypopnea index; CPAP, continuous positive airway pressure

Supplemental Table S1. FGM indexes of normal participants and patients with OSA (n=55)

| FGM indexes during sleep | Normal (n = 13) | OSA (n = 42) | p value |
| --- | --- | --- | --- |
| AG, mg/dL | 89.7 ± 5.9 | 100.3 ± 19.5 | 0.060 |
| SD, mg/dL | 7.9 ± 2.5 | 12.0 ± 5.4 | **0.004*** |
| CV, % | 8.7 ± 2.6 | 11.9 ± 4.0 | **0.009*** |
| TBR, % | 1.9 ± 2.8 | 5.8 ± 11.7 | 0.223 |
| TIR, % | 98.1 ± 2.8 | 91.9 ± 12.6 | **0.039*** |
| TAR, % | 0 | 1.1 ± 3.6 | 0.096 |
| LBGI, | 2.3 ± 1.2 | 2.5 ± 2.4 | 0.533 |
| HBGI | 0.2 ± 0.3 | 0.7 ±1.0 | **0.032*** |
| Magnitude of the dawn phenomenon, mg/dl | 12.6 ± 7.5 | 17.2 ± 8.3 | **0.040*** |
| FGM indexes during 24 h |  |  |  |
| AG, mg/dL | 103.5 ± 6.3 | 113.2 ± 19.2 | 0.088 |
| SD, mg/dL | 19.6 ± 3.5 | 25.0 ± 8.5 | **0.048*** |
| CV, % | 19.0 ± 3.3 | 22.0 ± 5.5 | 0.113 |
| TBR, % | 0.8 ± 1.5 | 2.7 ± 4.5 | 0.082 |
| TIR, % | 98.5 ± 1.6 | 91.9 ± 8.2 | **<0.001*** |
| TAR, % | 0.7 ± 0.8 | 4.9 ± 8.2 | **0.046*** |
| LBGI | 1.6 ± 0.9 | 2.0 ± 1.5 | 0.774 |
| HBGI | 1.3 ± 0.6 | 2.3 ± 1.8 | 0.080 |

Data are presented as mean ± SD

Statistical significance of differences in mean values between two groups was assessed with Mann-Whitney U test. * p values ≤ 0.05 were considered significant.

*FGM* flash glucose monitoring system, *OSA* obstructive sleep apnea, *AG* Average glucose, *SD* standard deviation, *CV* coefficient of variation, *TBR* time below range (percentage of time for which glucose level was <70 mg/dL), *TIR* time in range (percentage of time for which glucose level was 70–180 mg/dL), *TAR* time above range (percentage of time for which glucose level was >180 mg/dL), *LBGI* low blood glucose index; HBGI, high blood glucose index

Supplemental Table S2. Polysomnographic indexes in patients with OSA (n=42)

| Polysomnographic indexes |  |
| --- | --- |
| TST, min | 408.1 ± 96.9 |
| REM sleep time/TST, % | 13.0 ± 4.6 |
| N1 stage time/TST, % | 44.7 ± 22.8 |
| N2 stage time/TST, % | 39.9 ± 20.6 |
| N3+4 stage time/TST, % | 2.3 ± 3.9 |
| AHI, no/hr | 49.7 ± 31.0 |
| REM-AHI, no/h | 53.0 ± 21.3 |
| NREM-AHI, no/h | 45.8 ± 31.9 |
| ArI, no/h | 50.8 ± 29.8 |
| ODI, no/h | 44.2 ± 31.9 |
| SLT90, % | 16.2 ± 24.5 |

Data are presented as mean ± SD.

*OSA* obstructive sleep apnea, *TST* total sleep time, *REM* rapid eye movement, *AHI* apnea/hypopnea index, *NREM* non-rapid eye movement, *ArI* arousal index, *ODI* oxygen desaturation index, *SLT90* percentage of sleep time spent with oxygen saturatio
